# General practice incentives: a review of the international literature

**DOI:** 10.3399/BJGP.2025.0143

**Published:** 2026-04-08

**Authors:** Mark Harris, Joel Rhee, Margo Barr, Anurag Sharma, Ben Harris-Roxas, Mary Burns, Kate Marshall, Rula Ali, Anthony P Sunjaya, Cathelijne van Kemenade, Michael Kidd

**Affiliations:** 1 Faculty of Medicine and Health, International Centre for Future Health Systems, University of New South Wales, Sydney, Australia; 2 Faculty of Medicine and Health, School of Clinical Medicine, University of New South Wales, Sydney, Australia; 3 Faculty of Medicine and Health, School of Population Health, University of New South Wales, Sydney, Australia; 4 Evaluation and Evidence Services, Sax Institute, Glebe, Australia

**Keywords:** general practice, incentives, research methods, reform, primary health care, systematic review

## Abstract

**Background:**

In Australia, the general practice sector faces significant challenges, leading to a re-evaluation of its predominantly fee-for-service (FFS) funding models.

**Aim:**

To conduct a systematic review of reviews that evaluated the efficacy of funding models in general practice on quality outcomes in multidisciplinary primary and preventive care for people with complex chronic conditions, as well as the contextual factors that have influenced their implementation.

**Design and setting:**

Only systematic reviews and meta-analyses were included. Search terms covered funding mechanisms, primary care, and general practice. The review followed PRISMA guidelines for systematic reviews.

**Method:**

PubMed, Cochrane, Embase, CINAHL, PsycInfo, PAIS, and Web of Science databases were searched in November 2023 for publications from 2010 onwards.

**Results:**

Twenty reviews were included. Blended payment models incorporating pay-for-performance (P4P) with either capitation payments or FFS were associated with small improvements in quality outcomes, particularly in multidisciplinary settings. Changes in intermediate health outcomes and process measures for people with complex chronic conditions were most evident in diabetes care but inconclusive for other health outcomes and conditions. Improvements were mainly observed in incentivised activities and in less rigorously designed studies, with ceiling effects and variation reported across healthcare systems. There was no evidence that introducing capitation payments as part of blended payments improved quality of care.

**Conclusion:**

Although blended payment models show promise, evidence for the effectiveness of models including P4P is highly variable. If adopted, careful evaluation of each incentive’s impact on both quality and equity will be essential.

## How this fits in

Blended models of funding general practice combine elements such as fee-for-service, capitation, and performance-based payments to better support multidisciplinary care. Although internationally these models have shown potential, existing evidence has been mixed, with improvements most often limited to incentivised processes rather than patient-centred outcomes. The current review synthesised international literature to address gaps in understanding how different funding models influence prevention and chronic disease management. Although blended funding models may improve some care processes, there is currently no evidence on whether they affect time with GPs, waiting times, or continuity of care equity.

## Introduction

Blended models of funding general practice in countries around the world involve a combination of payment structures or funding sources to support primary care services. This approach often incorporates multiple elements, such as fee-for-service (FFS), in which primary care receives payments for each service provided or procedure performed. In Australia, despite the introduction of some blended payments, the current funding model is still predominantly based on FFS, and this may be less suited to supporting multidisciplinary prevention and management of long-term conditions because it does not adequately fund services that are not face-to-face or where there is a shared responsibility across the healthcare team.^1^

Capitation payments are an alternative to FFS, in which primary care services receive a set amount of funding per patient registered with their practice. Although capitation payments have been associated with improved measures of quality of care in some research,^2^ in others they have also been associated with reduced access and frequency of preventive care.^3,4^

Other funding models are based on performance-based payments (P4P). In these, incentives are tied to achieving specific healthcare targets or quality indicators. For instance, primary care services might receive bonuses for meeting certain clinical outcome goals, patient satisfaction levels, or participation in preventive health programmes. There are also other models, such as small grants or lump sum payments, which were recently provided on a one-off basis to Australian general practices to support innovation, training, equipment, and minor capital works to address some of the pressures on the system.^5^

A blended funding model aims to create a more comprehensive and flexible approach to support primary care services in delivering quality care while considering various aspects of patient needs, preventive care, and healthcare system goals. It is often intended to incentivise efficient, patient-centred care and to encourage better health outcomes;^6^ health outcomes being defined as measurable changes in an individual’s or population’s health status resulting from healthcare interventions, including clinical, functional, psychological, and social aspects of wellbeing.^5^ MyMedicare was launched in Australia at the end of 2023^7^ and introduced voluntary patient registration to strengthen the relationship between patients, their general practice, GP, and primary care teams. MyMedicare has been accompanied by additional FFS payments for longer consultations and telehealth consultations, and the introduction of a blended funding model designed to incentivise better quality of care. This differs from current Practice Incentive Program (PIP) and PIP Quality Improvement payments (which primarily provide activity-based payments for participation and data reporting) in that it combines FFS with capitation and quality-based payments to incentivise continuity and quality of care.

This study sought to address a gap in understanding how funding models affect prevention and multidisciplinary care in general practice, providing evidence to guide current Australian Strengthening Medicare reforms^5^ — including MyMedicare, new funding for multidisciplinary team-based care, and expanded use of blended payment models — aimed at improving continuity, quality, and equity of care through a systematic review of reviews.

The research questions were to determine the effectiveness of different payment models on:

- healthcare quality outcomes in multidisciplinary settings; and
- preventive care for people with complex chronic disease.

## Method

This review of systematic reviews was conducted in accordance with the Preferred Reporting Items for Systematic Reviews and Meta-Analyses (PRISMA) statement.^8^

Scott *et al*,^9^ in their Cochrane review of the literature between 2000 and 2009 on the effect of financial incentives on the quality of health care provided by primary care physicians, examined:

- different types of financial incentives that have improved quality;
- characteristics of patient populations for whom quality of care has been improved by financial incentives; and
- characteristics of primary care providers who have responded to financial incentives.^9^

The present review followed the same approach but focused on systematic reviews and meta-analyses, between January 2010 and November 2023.

The following databases were searched in November 2023 for publications since 2010: PubMed, Cochrane, Embase, CINAHL, PsycInfo, PAIS, and Web of Science. Search terms covered funding mechanisms, impact on primary care and general practice, and systematic review/meta-analysis. See Box 1 and Supplementary Information S1 for detailed search terms. Additional citation searches were conducted.

Box 1. **Description of search inclusions used**

**Types of studies**

- systematic reviews;
- meta-analyses; and
- key citations included in systematic reviews and/or meta-analyses.

**Types of participants**
Individuals and/or settings offering or receiving primary health care:

- general practice; and
- primary health care.

**Types of interventions**
Primary care and general practice incentives and funding models:

- capitated funding;
- performance incentives; and
- blended funding.

**Types of outcome measures**
Primary outcome measures include the quality of care provided by primary care providers that are related to patients’ health and wellbeing:

- access to care;
- quality of care;
- preventive care;
- multidisciplinary care;
- health outcomes;
- cost of health care; and
- provider behaviour.

The resultant literature from each of the searches were loaded into Covidence 2023. Covidence was used to manage the screening and extraction of data from the literature. Duplicates were identified and removed. Eligible articles needed to be systematic reviews or meta-analyses assessing health outcomes of funding mechanisms in primary care settings in high-income countries. Two reviewers independently screened the titles and abstracts using the inclusion and exclusion criteria (see Supplementary Box S1). Articles were excluded if they only included specialist or hospital services, only included patient payments, were not in English, or only included low- or middle-income countries. The full text of the selected articles was then reviewed by two members of the team. Any discrepancies were discussed and resolved by the reviewers. If disagreements could not be resolved, a third reviewer was asked to resolve it.

Data abstraction was conducted using a modified version of the PRISMA checklist for systematic reviews.^10,11^ Items extracted covered objectives, methods of screening, data extraction, quality assessment and synthesis, results, limitations, and interpretation (see Supplementary Boxes S2 and S3). Data extraction was checked by a second reviewer.

The quality of each included systematic review article was evaluated using the ROBIS (Risk Of Bias In Systematic reviews) tool.^12^ ROBIS is designed to assess the risk of bias (RoB) in systematic reviews with questions relating to interventions, aetiology, diagnosis, and prognosis. It assesses four domains from which bias may be introduced: 1) study eligibility criteria; 2) identification and selection of studies; 3) data collection and study appraisal; and 4) synthesis and findings.

For each publication, the researchers also examined heterogeneity, methods, funding models, and settings. Outcomes analysed included access to care, quality of care, preventive care, multidisciplinary care, health outcomes, cost of health care, and provider behaviour.

Evidence was categorised as ‘sufficient’ or ‘insufficient’ based on the number, quality, consistency, and relevance of included reviews; sufficient evidence required multiple high- or moderate-quality reviews with consistent findings, whereas insufficient evidence reflected few studies, conflicting results, or limitations in quality or relevance.

## Results

**Figure 1.**
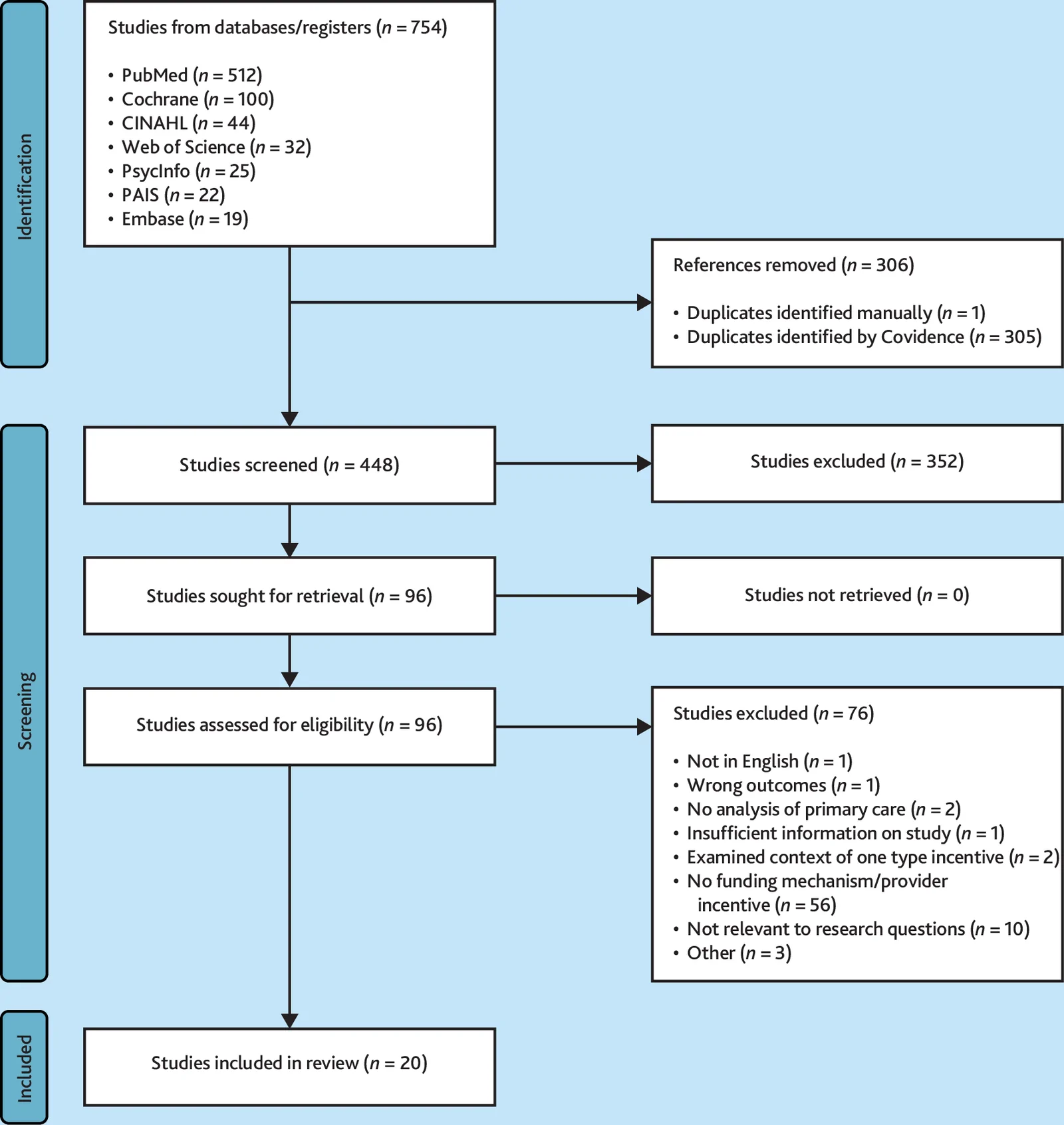
PRISMA flowchart of systematic review selection process.

The systematic search revealed 754 publications. Removing duplicates left 448 publications, of which the title and abstract were screened. Ninety-six articles were full-text assessed; 20 fulfilled the criteria for inclusion. The PRISMA flowchart is shown in Figure 1.

### Description of studies

The systematic search resulted in the inclusion of 14 systematic reviews, three meta-analyses, two reviews of reviews, and one other review (see Supplementary Box S4).^13–32^ The majority of studies evaluated schemes in the UK (*n* = 16), US (*n* = 13), Europe (*n* = 9 across Denmark, Finland, Sweden, Germany, Netherlands, Belgium, Italy, Spain, France, and Ireland), and Canada (*n* = 9). Six included studies were conducted in Australia. A smaller number of studies included in the reviews originated in Asia-Pacific (New Zealand, Taiwan, Japan, and South Korea). See Supplementary Boxes S5 and S6 for more details about each study.

The focus of the systematic search was on general practice, but some of the eligible studies were set in other related primary healthcare settings including dentistry, allied health, nursing, and mental and community health. The health areas the studies targeted covered chronic disease, diabetes, cardiovascular disease, respiratory disease, mental illness, lower back pain, smoking cessation, cancer screening, and laboratory tests and processes.

The main payment scheme assessed across the reviews was P4P (*n* = 20), but also included were FFS, capitation payments, bonus payment, mixed, and other models. The aim of the interventions was to improve the quantity and/or quality of health service provision through, for example, the implementation of indicators, changing provider behaviour, or retention of medical workforce.

### Quality assessment of included systematic reviews

**Figure 2.**
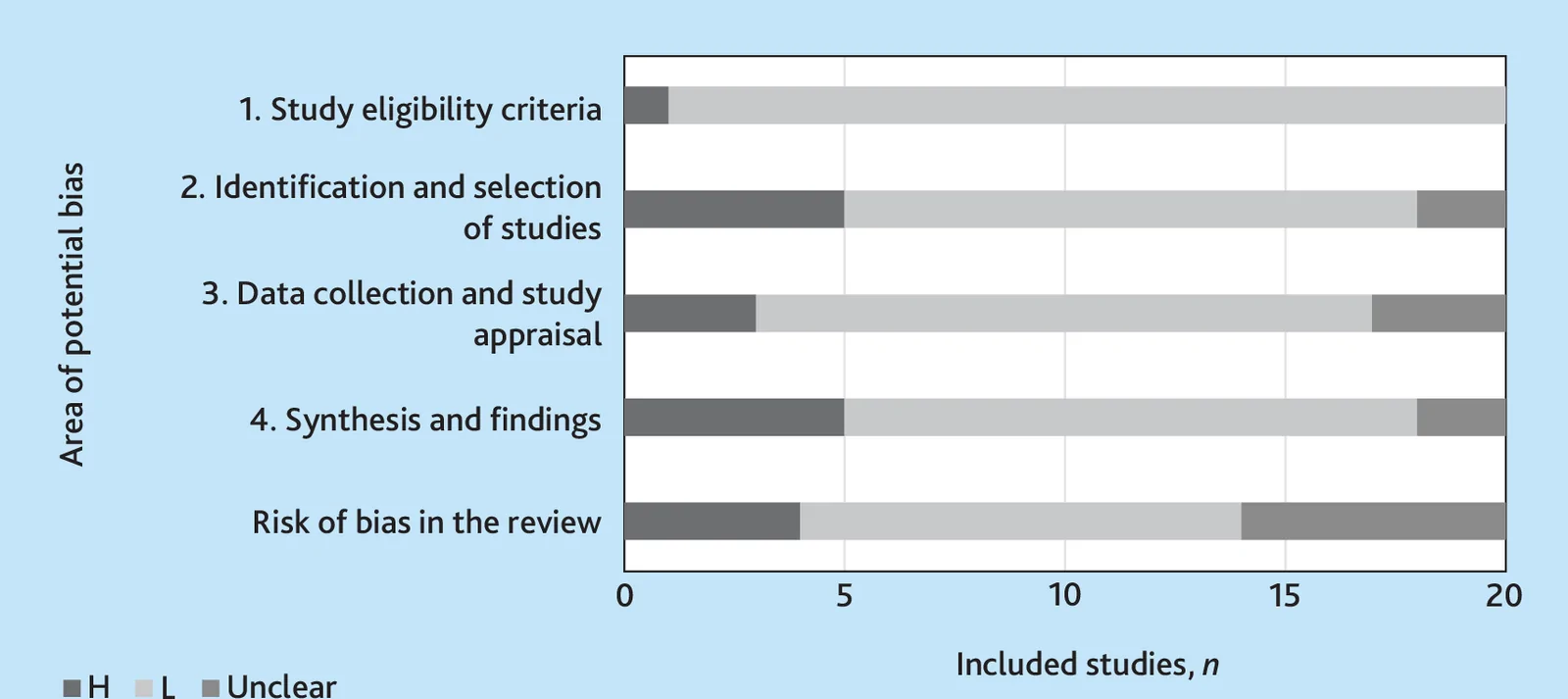
Overall assessment of risk of bias assessment. H = high. L = low.

As shown in Figure 2, RoB were provided (high, low or unclear) for each of the areas of potential bias introduced during the review process (study eligibility criteria; identification and selection of studies; data collection and study appraisal; and synthesis and findings) as well as overall. For the overall RoB outcome assessments the majority of articles (50%, *n* = 10) were categorised as low RoB. Summary details by article are provided in Box 2.^13–32^ The individual responses to the relevant ROBIS signalling questions for each domain that underlie the summary assessment are provided in Supplementary Box S7 to justify the quality ratings provided in Box 2.

**Box 2.** Assessment of risk of bias assessment for each systematic review

Study ID	1. Study eligibility criteria	2. Identification and selection of studies	3. Data collection and study appraisal	4. Synthesis and findings	Overall risk of bias
**Ahmed (2021)** ^13^	Low	Low	Low	Unclear	Unclear
**Boeckxstaens (2011)** ^14^	Low	Low	Unclear	Low	Low
**Carter (2016)** ^26^	Low	High	Low	Low	Unclear
**Emmert (2012)** ^15^	Low	Low	Low	Low	Low
**Flodgren (2011)** ^16^	Low	Low	Low	Low	Low
**Gillam (2012)** ^17^	Low	Low	Low	Low	Low
**Hamilton (2013)** ^25^	Low	High	Low	High	High
**Heider (2020)** ^18^	Low	Low	High	Low	High
**Houle (2012)** ^27^	Low	Low	Low	Low	Low
**Jia (2021)** ^19^	Low	Low	Low	Low	Low
**Langdown (2014)** ^28^	Low	Unclear	Unclear	High	Unclear
**Lin (2016)** ^29^	Low	High	Low	High	High
**Mandavia (2017)** ^20^	High	Low	Low	High	Unclear
**Mauro (2019)** ^30^	Low	Low	High	Unclear	Low
**Ogundeji (2016)** ^21^	Low	Unclear	Low	Low	Unclear
**Scott (2018)** ^22^	Low	High	High	High	High
**Tao (2016)** ^23^	Low	High	Unclear	Low	Unclear
**Van Herck (2010)** ^24^	Low	Low	Low	Low	Low
**Wranik (2019)** ^31^	Low	Low	Low	Low	Low
**Yuan (2017)** ^32^	Low	Low	Low	Low	Low

### Research question 1: impact on healthcare quality outcomes in multidisciplinary settings

Fourteen reviews provided evidence of the impact on healthcare quality of care.^13–24,31,32^ There was sufficient evidence that blended payments involving P4P in combination with capitation payments or FFS were likely to bring about small improvements in quality of care. This was more pronounced if the performance measure was an intermediate outcome (such as, haemoglobin A1c) or a process of care measure (such as, recording of risk factors) rather than outcome measures or patient experience.^14,16–21,24^

There was some evidence that P4P had a greater impact on quality of care when it was combined with non-financial incentives (such as, reputational) and quality improvement.^9,24^ There was insufficient evidence for improvement in health outcome measures. For example, in Hamilton *et al*,^25^ improvements were noted in quality of care and process measures from P4P but not for smoking quit rates. Changes in smoking prevalence could be attributed to causes other than doctor management. In Jia *et al* the authors found uncertain effects of P4P on vaccination rates and blood pressure control in older adults.^19^

There was no evidence that either capitation payments or P4P reduced health inequalities.^23^ There were some mixed qualitative and quantitative findings suggesting that care that was not incentivised did not improve, and that targets may be achieved at the expense of holistic person-centred care.^13^ There was some evidence that incentives, especially where funds were pooled at the practice level, supported teamwork and collaboration between primary care providers and disciplines.^17,24,31^

The quality of the reviews was variable, with those with low RoB being less likely to show positive effects. Most of the research on quality of care in multidisciplinary primary care settings was from the UK, US, and Canada. Several contextual factors need be taken into account, including:

- how benefits are distributed between organisations and providers and between individual providers and teams; and
- the ability of primary care providers (and groups of their patients) to include or exclude some patients from enrolment (this effect could be minimised by encouraging rostering to ensure continuity of care), as this may distort the effects on performance and thus funding.^14^

### Research question 2: impact on preventive care for people with chronic conditions

Ten reviews were eligible for inclusion.^13,23–30,32^ Six reviews reported the types of complex chronic illnesses in patients, including diabetes, stroke, asthma, hypertension, severe mental illness, cardiovascular diseases, and respiratory diseases.^23,24,26,28,29,32^ Four reviews included studies reporting on preventive care services (such as, cancer screening, immunisations, or smoking cessation assistance) for the general patient population.^25,27,30,32^ Five reviews examined the impact of quality-based payment incentives on equity of chronic disease care.^13,23,24,26,29^ Overall, findings were mixed and variable, with some reviews reporting a positive impact on health equity, whereas others showed a neutral or negative impact.

There was insufficient evidence of the impact of P4P or financial incentives in general practices on hard health outcomes (for example, mortality rates), particularly among patients with complex chronic disease, owing to the scarcity of research focusing on primary health outcome measures. For example, one review included only a single hypertension study that examined end-organ complications, such as heart failure, acute myocardial infarction, stroke, and renal failure.^19^ Another review highlighted that only one of the five clinical indicators in the UK’s Quality and Outcomes Framework (QOF) was a health outcome indicator.^28^

Some studies indicated positive effects of P4P or financial incentives on preventive care activities, process measures, and intermediate health outcomes, particularly in patients with diabetes.^24,25,27,28,30,32^ However, these findings varied widely across studies, disease types, outcome measures, and programme designs. A positive impact, where present, tended to be small, and improvements were mostly confined to incentivised activities, sometimes negatively affecting non-incentivised areas.^24,27,28,32^ There was an observed ceiling effect in improvements, plateauing after reaching the maximum thresholds set for financial incentives in programme implementation.^13,23,24,28^

## Discussion

### Summary

This review of the international literature was undertaken to address gaps in existing systematic literature reviews by synthesising evidence on how funding models influence prevention and multidisciplinary care in general practice. It provides new insights into the contextual factors affecting implementation, variations in effectiveness across settings, and implications for current Australian Strengthening Medicare reforms aimed at improving continuity, quality, and equity of care for patients with complex chronic conditions. This review found limited evidence that blended payment systems including P4P improve outcomes in multidisciplinary or preventive care for patients with chronic conditions. Incentives under P4P or FFS may encourage providers to increase services for activities that are rewarded, potentially at the expense of non-incentivised and patient-centred care. Improvements in quality of care associated with such incentives appear to vary depending on context and on design features, such as how performance is defined and measured.^19,28,33^

The current review found that there was sufficient evidence on the effectiveness of incentives in changing process measures for quality of care. This may partially relate to the quality and availability of data to assess performance using these measures. The impact of incentives linked to these performance indicators was found to vary depending on the indicators used. Studies reported that measures, such as those used in the UK QOF, were more focused on processes of care, such as recording of assessments, but were not effective in improving adherence to high-value care, as per guidelines, or in optimising health outcomes.^16,18,21,28^ This focus on recording process measures for incentivised care may have contributed to a reduction in the interactions between providers and patients.^13^ This suggests that new performance frameworks should include patient-centred care measures — such as shared decision making, continuity, and patient satisfaction — while minimising the real-time documentation burden on providers, for example, through streamlined or retrospective data collection, to monitor quality without adversely affecting provider–patient interaction.^13^ Few reviews^17,31^ included direct patient perspectives or qualitative accounts; patient-centred measures were largely proxy indicators rather than first-hand patient experiences, highlighting an important gap for future research.

The current review found evidence that when there were performance-based and blended models, physicians were more likely to self-select to deliver services that were FFS or incentivised.^19^ There was also evidence that performance-based funding models may positively influence their provision of preventive care, both for primary and secondary prevention, although results varied across studies, diseases, and programme designs.^25^ Considering this, in blended capitation models it can be beneficial to link preventive and other high-value services to incentives so as to promote their delivery, while including low-value services under capitation agreements to reduce their unnecessary utilisation. It is also important to have clear criteria for inclusion to prevent distortions because of selective inclusion of practitioners and patients.^9^

The variability in the impact on quality and cost-effectiveness of performance-based and blended funding models between settings, and within the same system for different conditions, suggests that improvements in designing performance indicators and incentives alone may be insufficient. Economic evaluations should therefore be undertaken for each performance indicator particularly before full implementation.^34,35^ Main costs of an indicator could be costs associated with additional GP and nurse consultations for regular monitoring of patients’ health status, initial setup costs of implementing the intervention, and any additional usage of services such as diagnostic services. Regarding cost-effectiveness, blended payment models in primary care can influence physician behaviour and improve care quality, but they are often costly and not always cost-effective.^15,19,24^ Evidence suggests that although some incentive programmes reduce unnecessary services and admissions to hospital,^21^ others (like the UK’s QOF) may require redesign to justify their expense,^17,20,28^ highlighting the need for indicator-level economic evaluations before implementation.^15,29^

Although there was evidence that the UK’s QOF reduced the gap in quality of care between the most and least deprived areas,^13^ there was insufficient evidence on the impact of performance-related pay on health equity (defined here as avoidable disparities in care or outcomes between population groups).^13,15,23,24,29^ There was limited evidence for ceiling effects because of thresholds set by performance indicators, such as, delivery of a particular service to between 50% and 90% of the eligible cohort.^28^ However, raising thresholds to 100% may unfairly disadvantage facilities in deprived areas, as they would be less likely to reach targets.^28^ There is also a need for more studies reporting disaggregated results by gender and other equity groups (such as socioeconomic status and rurality).^15^

This review did not specifically examine the impact of various funding models on different members of the multidisciplinary team or on workforce supply and arrangements. However, some evidence indicates that when performance-related payments or capitation payments were implemented at a practice level, there was greater collaboration between primary care providers and other disciplines.^31^ These findings suggest that funding models that move beyond FFS may support team-level reforms by promoting coordinated care rather than focusing solely on individual service provision. Linking incentives to providers or teams as well as to other capacity development activities, such as training and quality improvement, may enhance the impact of these incentives. Modelling should be undertaken to ascertain the impact of any shift in funding models and overall investment in health care on workforce supply and interactions.

### Strengths and limitations

The strength of this review was that it synthesised evidence across multiple systematic reviews, which has clarified the effectiveness and contextual factors of these models, identifying gaps regarding health outcomes, costs, and equity, and has highlighted the potential synergistic role of combining financial and non-financial incentives. It also provides evidence to address gaps in understanding about how different funding models influence prevention and chronic disease management, with relevance for current Strengthening Medicare reforms, including MyMedicare.

Regarding the limitations of this study, reviewing systematic reviews of previous studies creates a time lag between the date of an intervention and the evidence provided in this report. Therefore, this review was likely to exclude any studies relating to changes in provider behaviour associated with the COVID-19 pandemic, which has offered opportunities for pre–post style reviews of various reforms in primary healthcare practice.

Also, few of the included studies focused on provider behavioural change as a primary outcome rather than as an enabling factor of changes in patient outcomes. The included studies were of low-to-moderate levels of quality and assessed as having moderate-to-high RoB. Relatively few controlled trials or natural experiments have been undertaken, owing to the interventions being primarily related to policies and financing arrangements, which does not lend itself to trial, pre–post, or comparative study designs. As such, there was generally limited information on the comparative effectiveness of specific measures.

Although a comprehensive search was undertaken across multiple databases, some relevant studies may have been missed, including unpublished or grey literature, non-English publications, or studies in non-indexed journals, introducing potential language and publication bias. In addition, variations in reporting and methodology across the included reviews may have limited the comparability and generalisability of the findings. The current review may have inadvertently excluded evidence on disadvantaged groups because of inconsistent reporting in the literature. This highlights a priority area for future research. Also, this review found limited research that directly captured the voice of patients regarding funding models.^17,31^ Most studies focused on process or intermediate outcomes, highlighting a clear gap. Future research should include patient perspectives and outcomes to understand the real-world impact of funding reforms on care quality and experience.

### Comparison with existing literature

Previous systematic reviews of payment reforms in primary care have reported mixed effects on quality and utilisation outcomes.^16,36^ Consistent with earlier evidence, the current review found that alternative payment models such as capitation, bundled payments, and pay-for-performance can improve selected process measures — particularly when incentives support team-based care — but have less consistent effects on patient outcomes.^9,37,38^ Unlike most prior reviews, which focused on single model types or physician-level incentives, the current synthesis highlights that multidisciplinary alignment of financial incentives appears critical to achieving sustained quality gains.^31^

### Implications for research and practice

Although various blended funding models have been implemented in many countries, it is important to tailor them to the local context (for example, Australia) because of the variability of impact for the same models in different settings. One important consideration in Australia is the impact on workforce supply, especially in underserved areas. Although this was outside the scope of this review, any changes in the mix of blended funding models needs to consider the likely impact on health workforce distribution.

A key element in the implementation for performance-based or blended models is the development of ‘fit for purpose’ indicators that combine process and health outcome measures. A careful evaluation of each individual performance measure needs to be undertaken. This evaluation should be based on how the effects vary according to context, scope for improvement, benefit, and cost of the measure, and should include both the positive and negative effects.

In conclusion, there was insufficient evidence that a shift to include more performance-based incentives in blended primary care funding models would result in improvements in health outcomes and minimise costs. There was sufficient evidence that linking incentives to not only performance indicators but also non-financial incentives, such as quality improvement programmes, may synergistically improve outcomes.

## Data

All data relevant to this systematic review are included within the manuscript and its supplementary materials.
